# Collagen XV Inhibits Epithelial to Mesenchymal Transition in Pancreatic Adenocarcinoma Cells

**DOI:** 10.1371/journal.pone.0072250

**Published:** 2013-08-22

**Authors:** Anthony G. Clementz, Michael J. Mutolo, Shih-Hsing Leir, Kirsten J. Morris, Karolina Kucybala, Henry Harris, Ann Harris

**Affiliations:** 1 Human Molecular Genetics Program, Lurie Children’s Research Center, Chicago, Illinois, United States of America; 2 Department of Pediatrics, Northwestern University Feinberg School of Medicine, Chicago, Illinois, United States of America; 3 Robert H. Lurie Comprehensive Cancer Center, Northwestern University Feinberg School of Medicine, Chicago, Illinois, United States of America; 4 Sir William Dunn School of Pathology, University of Oxford, Oxford, United Kingdom; Indiana University School of Medicine, United States of America

## Abstract

Collagen XV (COLXV) is a secreted non-fibrillar collagen found within basement membrane (BM) zones of the extracellular matrix (ECM). Its ability to alter cellular growth *in vitro* and to reduce tumor burden and increase survival *in vivo* support a role as a tumor suppressor. Loss of COLXV during the progression of several aggressive cancers precedes basement membrane invasion and metastasis. The resultant lack of COLXV subjacent to the basement membrane and subsequent loss of its interactions with other proteins in this zone may directly impact tumor progression. Here we show that COLXV significantly reduces invasion of pancreatic adenocarcinoma cells through a collagen I (COLI) matrix. Moreover, we demonstrate that epithelial to mesenchymal transition (EMT) in these cells, which is recapitulated *in vitro* by cell scattering on a COLI substrate, is inhibited by over-expression of COLXV. We identify critical collagen-binding surface receptors on the tumor cells, including the discoidin domain receptor 1 (DDR1) and E-Cadherin (E-Cad), which interact with COLXV and appear to mediate its function. In the presence of COLXV, the intracellular redistribution of E-Cad from the cell periphery, which is associated with COLI-activated EMT, is inhibited and concurrently, DDR1 signaling is suppressed. Furthermore, continuous exposure of the pancreatic adenocarcinoma cells to high levels of COLXV suppresses endogenous levels of N-Cadherin (N-Cad). These data reveal a novel mechanism whereby COLXV can function as a tumor suppressor in the basement membrane zone.

## Introduction

Unlike organized fibrillar collagens, which are abundant in the extracellular matrix (ECM), non-fibrillar collagens have only recently been associated with early events in cancer progression. Type XV collagen (COLXV) belongs to the non-fibrillar multiplexin collagen family [1], which unlike fibrillar collagens have interruptions within their collagenous domain [2], [3]. COLXV was first isolated from a human placental cDNA library and is encoded by the *COL15A1* gene on chromosome 9q21 [4], [5], [6]. The hypothesis that COLXV might be a tumor suppressor was first proposed in 2003 [7], based on cytogenetic analysis of tumorigenic segregants of somatic cell hybrids in which malignancy was suppressed. Reversion of malignancy was accompanied by consistent loss of a small region of mouse chromosome 4 and disappearance of secreted extracellular matrix. The chromosome 4 fragment was subsequently shown to encompass the mouse COLXV gene and to be syntenic with a region of human chromosome 9. COLXV is a secreted 1388 amino acid protein localized within the outermost lamina densa in basement membrane zones of the ECM [8]. The protein encompasses three regions: the N-terminal non-collagenous domain, the central collagenous region, and the C-terminal related to endostatin (restin) domain that shares some homology with cleaved endostatin in collagen XVIII (COLXVIII) [9]. However, unlike endostatin, the restin domain alone does not have tumor suppressive properties *in vitro* or *in vivo* [10]. The molecular interaction of native COLXV with other BM and ECM components and surface receptors is not well characterized. Loss of COLXV during tumor progression in aggressive breast tumors, colon carcinomas and melanomas precedes invasion and metastasis [11], [12], [13]. COLXV null mice (*Col151a −/−*) show aberrant matrix biology in multiple organs, implicating a role for this protein in tissue remodeling events, protein interactions, and molecular signaling [14], [15]. COLXV is distinct from the many fibrillar collagens that are involved in cancer progression due to its unique location within basement membrane zones (the first areas compromised in tumor extravagation), and its flexible pretzel-like structure, in contrast to the rigid fibrillar collagens such as collagen I (COLI). Moreover, during cancer progression, fibrillar collagens contribute to characteristic fibrosis while non-fibrillar COLXV is lost prior to metastasis. The associated loss of structural integrity of the basement membrane zone likely contributes to poor survival since it may facilitate invasion, initially through the basement membrane, leading to distal metastases.

In addition to the stromal alterations, tumor cells themselves carry multiple classes of cell surface receptors that show altered expression and activity during cancer progression. These include discoidin domain receptors (DDRs) and integrins among others that interact with the microenvironment. DDRs are a family of type I transmembrane receptor tyrosine kinases that actively bind to interstitial collagens, including COLI, within the ECM and mediate stroma/cellular signaling. DDRs can interact with both fibrillar and non-fibrillar collagens in the extracellular matrix [16], [17]. COLI binding activates auto/trans-phosphorylation of DDR and ectodomain shedding via disintegin and metalloproteases (ADAMs) [18]. In turn Pyk2, a downstream target of DDR1 is also phosphorylated activating a well-characterized signalling cascade [19]. DDR1 is overexpressed in many cancers, associated with cell proliferation [13], migration, metastasis and development [20], [21], [22], [23], [24]. Integrins (α_n_ and β_n_) are a heterogeneous family of collagen-binding transmembrane proteins that are intimately involved in stroma/cellular crosstalk. Although they lack a kinase domain, their activity involves a complex array of downstream adaptor proteins, such as focal adhesion kinase (FAK), which themselves are phosphorylated [25]. The integrins and their combinations, notably α_2_β_1_-integrin, demonstrate functional differences depending on cellular response to their environment. For instance, inhibition of β_1_-integrin reduced the aggressive phenotype of human breast tumor cells, and targeted deletion of β_1_ integrin in the mammary epithelium *in vivo* prevented tumor initiation and growth [26]. Independent shRNA-mediated knockdown of β_1_ integrin and to a lesser extent α_2_ integrin reduced primary growth and metastasis in an orthotopic pancreatic adenocarcinoma mouse model [27]. In contrast, previous studies suggested that loss of α_2_β_1_ integrin may facilitate cancer progression [28]. Moreover, *in vivo* studies in mice and human clinical studies demonstrated that loss of α_2_β_1_ integrin may predict metastasis and decreased survival rates in cancer, thus implicating a potential role for α_2_β_1_ integrin as a metastasis suppressor [29]. Thus integrins may have opposite roles in tumor progression depending on cellular content and environmental factors.

E-Cadherin (E-Cad) is another important cell adhesion protein associated with tumor growth, it is calcium dependent and shows loss of expression and/or re-localization during tumor progression [30]. The majority of E-Cad localizes to adherens junctions, but the protein is also found throughout polarized cells in both apical and basolateral zones [31]. Degradation of E-Cad occurs via endocytosis targeted to the proteasome (reviewed in [32]). During the process of epithelial to mesenchymal transition (EMT) E-Cad expression is either greatly reduced, or its location is altered as it associates with early endosomes in the cytosol and moves away from the cell periphery. In contrast, N-Cadherin (N-Cad) is greatly upregulated during EMT. Crosstalk between membrane receptor tyrosine kinases (RTKs) [33], E-Cad and additional factors including soluble ligands in the microenvironment may play a vital role in the stimulation/inhibition of classical signaling pathways aberrant in cancer.

COLXV is normally present in the stroma and its loss is evident preceding tumor progression, hence we tested the hypothesis that its interactions with cell-surface receptors such as α_2_β_1_ integrin, DDR1 and E-Cad, and subsequent signaling events might be critical to its function as a tumor suppressor. Moreover, that loss of COLXV from the basement membrane zone influences tumor/stromal cross-talk and facilitates EMT. Our hypothesis was based on earlier observations, by others [19], which showed that simultaneous interactions of DDR1 and α_2_β_1_ integrin with COLI, cause upregulation of N-Cad and cell scattering (EMT) in pancreatic cancer cells. Here we aimed to investigate whether COLXV interacts directly with these receptors and thus potentially disrupts EMT.

Since it is extremely difficult to generate recombinant COLXV that is correctly glycosylated and folded into its natural conformation, in heterologous expression systems, we instead produced the protein in the pancreatic adenocarcinoma cells BxPC-3 and S2-013. Mature COLXV is secreted from these cells into the culture media, enabling investigation of its effects on the behavior of the cancer cell lines when delivered from the outside, as would be the case within the basement membrane zone of the intact pancreatic duct. Moreover, we chose the BxPC-3 cell line as it demonstrates a dramatic scatter/EMT phenotype when grown on COLI substrates [19] making it a particularly suitable model to study EMT in pancreatic cancer.

We first show that overexpression of COLXV in pancreatic adenocarcinoma cells diminishes their ability to invade/migrate through a COLI matrix in comparison to vector control cells. Next, we determine that when COLXV is exogenously overexpressed in pancreatic adenocarcinoma cells and secreted into the medium, not only is cell scattering inhibited, but COLI-mediated redistribution of E-Cad from the cell periphery to the cytoplasm is also repressed. We show that DDR1 and E-Cad interact with COLXV and that the presence of COLXV in the extracellular milieu inhibits COLI - mediated DDR1 signaling. Moreover, although cell scatter-associated upregulation of N-Cad is not prevented by the presence of COLXV, chronic exposure to this protein reduces basal levels of N-Cad and this in turn reduces the total amounts of N-Cad at scatter. These data demonstrate a novel mechanism to prevent EMT and suggest a critical role for collagen XV in E-Cad stabilization and in modulating crosstalk between receptors that are central to EMT and tumor progression.

## Materials and Methods

### Cell Culture and Reagents

BxPC-3 were obtained from ATCC. S2-013 cells [34] were kindly donated by Dr S. Taniguchi. Scrambled siRNA-A (sc-37007), E-Cadherin siRNA (sc-35242) and DDR1 siRNA (sc-35187) were from Santa Cruz Biotechnology. Transfection reagents for siRNA delivery were Lipofectamine 2000 and Lipofectamine RNAiMAX (Invitrogen).

### Antibodies

COL15A1 (sc-16515), DDR1 (sc-532), E-Cadherin (sc-7870), N-Cadherin (sc-59987), pFAK 397 (sc-11765-R), FAK (sc-588), EEA1 (sc-6415) and DAPI (sc-3598) were from Santa Cruz Biotechnology. pPyk2 (44636G) was from Life Technologies and Pyk2 (06559) from Millipore. Anti-FLAG M2 (F3165) and β-tubulin (T4065) were from Sigma-Aldrich. Alexa-Fluor secondary antibodies were from Jackson ImmunoResearch Laboratories.

### Western Blot Analysis and Immunoprecipitation (IP)

The cells were lysed in RIPA buffer pH 8.0 or NET buffer by standard protocols and western blotting was performed as previously described (Harris 2007). For IPs lysates were incubated with DDR1 antibody, isotype control, or M2 conjugated agarose beads (A1220, Sigma Aldrich,) and DDR1 complexes were precipitated with protein G-plus beads (sc-2002, Santa Cruz Biotechnology). Western blots of IP proteins were probed with antibodies for DDR1, E-Cad, COLXV and FLAG.

### Protein Quantitation

Western blots probed with β-tubulin and the levels of FAK, Pyk2 and N-Cad measured relative to it. For FAK, pFAK, Pyk2, pPyk2 and N-Cad signals, the integrated density was quantified with the Lasso tool of Adobe Photoshop Extended CS5.

### Collagen I Scatter Assays

Scatter assays were established as described previously [19] using 50 µg/mL collagen I solution from rat tails (C3867-1VL, Sigma-Aldrich). The scatter phenotype was observed after 48–72 hr using a Leica DM-IRB Inverted Research Microscope, phase contrast at 100X magnification.

### Immunofluorescence

BxPC-3 scatter assays were established as described above. The E-Cad antibody recognized the extracellular domain of the protein and the secondary antibody was Alexa-Fluor 488 goat anti-rabbit IgG (111-545-003) with DAPI counterstain. For dual imaging the same E-cad antibody was used with an EEA1 antibody recognizing the N-terminus of the protein. Secondary antibodies were Alexa-Fluor 488-conjugated donkey anti-rabbit (711-545-003) and Alexa-Fluor 594-conjugated donkey anti-goat IgG (705-585-003). Confocal microscopy utilized a Zeiss 510 META Confocal Laser Scanning Microscope.

### Flow Cytometry

Flow cytometry was performed by standard methods using a FACSCalibur and N-terminal E-Cad antibody (sc7870). Data were analyzed by Cyquest software.

### Invasion Assays and Cell Doubling Time

The assay was as previously described [35], with modification [36]. Cells were plated in the upper chambers of the Membrane Invasion Culture System (MICS) apparatus on a porous membrane coated with gelatin and with COLI alone or COLI, COLIV and laminin in combination. Seventy-two hours later, the membrane was fixed and stained with Coomassie blue and noninvasive cells removed from the upper side of the membrane with a cotton swab. Invaded cells attached to the lower side of the membrane were counted from 6 random fields in triplicate and the final data set was from three independent experiments. *In vitro* growth rates of clones were determined using the CellTiter96 Aqueous Cell Proliferation Assay (MTS) (Promega), at 24, 48 and 72 hours after plating 7×10^3^ cells onto 96-well plates in triplicate.

## Results

### Generation of Stable Cell Lines in BxPC-3 and S2-013 Cells

**Figure 1:**
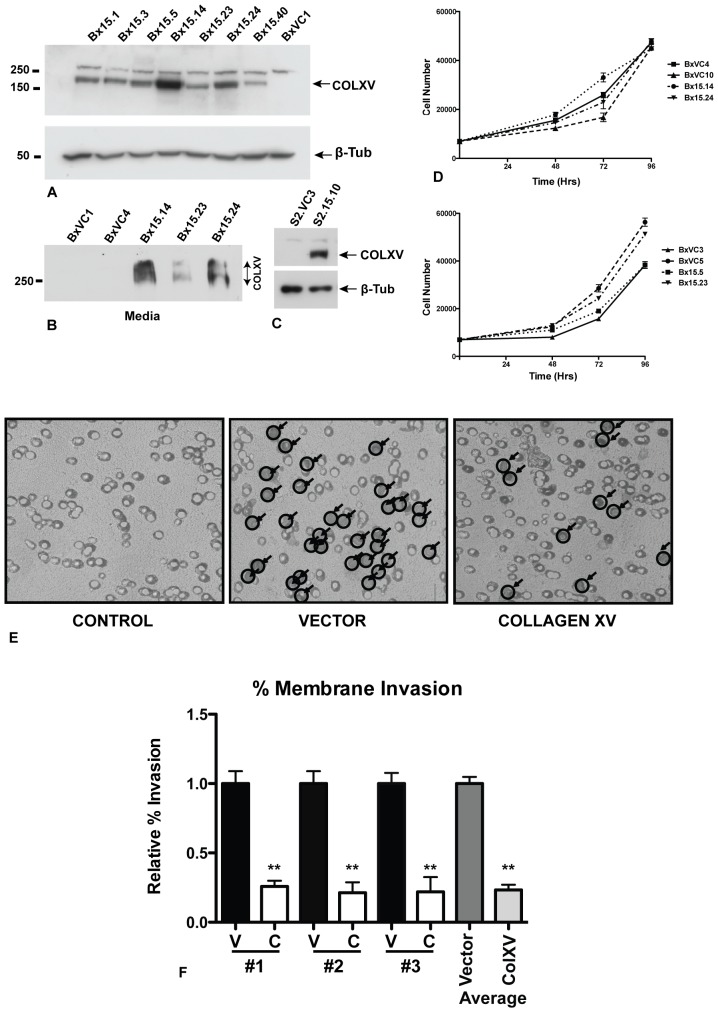
Collagen XV inhibits invasion of BxPC cells though a collagen I-coated membrane. A) Stable expression of collagen XV in BxPC-3 and S2-013 pancreatic adenocarcinoma cells. Western blot probed with anti-COLXV antibody or anti-ß-tubulin loading control. Clones Bx15.1-15.40 express variable levels of COLXV. Also shown is one vector control clone (BxVC1). Note: the anti-COLXV antibody cross-reacts with an irrelevant higher MW protein that is seen in many cell types, irrespective of COLXV expression. B) The COLXV is secreted from BxPC-3 cells into the cell culture media. C) COLXV expression in a representative stable clone of S2-013 cells (S2.15.10), also shown is a vector control (S2.VC3). D) Growth rates of 4 BxPC-3 vector control clones and 4 COLXV-expressing clones are independent of COLXV expression. E, F) Invasion assay: E) vector (BxVC1, BxVC3) or COLXV (Bx15.14 and Bx15.23) cells plated on a membrane coated with COLI. After 72 hr cells invading through the membrane were stained, visualized and counted at 40X magnification. Random fields were selected from three independent experiments representing n = 16 vector plates (BxVC1, BxVC3) and n = 15 COLXV plates (Bx15.14 and Bx15.23). F) The average of each independent experiment is shown #1, #2, #3, (Left, V = vector control; C = COLXV) and the cumulative average of all triplicates (right).

To investigate the mechanism of action of COLXV in modulating pancreatic tumor cell growth *in vitro*, we stably expressed a human COLXV cDNA construct in the BxPC-3 and S2-013 pancreatic adenocarcinoma cell lines. Vector (pcDNA3.1) control clones of each cell line were also generated. Unlike most pancreatic adenocarcinoma cell lines, BxPC-3 has wild-type KRas, while S2-013 carries the common KRas^G12D^ mutation. However, unlike S2-013, BxPC-3 demonstrates a marked scatter/EMT phenotype when grown on COLI substrates [19] and is thus an excellent model to study EMT in pancreatic cancer. Multiple clones were produced with a range of COLXV expression and 4 clones with high levels of COLXV were analyzed further (Bx15.5, Bx15.14, Bx15.23 and Bx15.24, Fig. 1A). Moreover, we showed that a substantial amount of COLXV is secreted from these clones into the cell culture medium (Fig. 1B). An equivalent series of cell clones expressing the COLXV transgene was generated from the S2-013 pancreatic adenocarcinoma cell line (Fig. 1C) and high-expressing clones S2.15.5 and S2.15.10 were taken forward for further analysis together with vector control clones.

### Pancreatic Cells Expressing Collagen XV Show a Reduction in Cellular Invasion

To determine whether COLXV altered the ability of pancreas cancer cells to invade the basement membrane we used an *in vitro* model of invasion through porous membranes [35]. BxPC-3 cells expressing COLXV or vector controls were plated in the upper chambers of the Membrane Invasion Culture System (MICS) apparatus on a porous membrane coated with gelatin and with COLI alone or COLI, COLIV and laminin in combination. Seventy-two hours later, the membrane was fixed, stained with Coomassie blue and invaded cells attached to the lower side of the membrane were examined by inverted light microscope. This method [36] was used rather than counting the numbers of cells that migrated into the lower chamber, as BxPC-3 cells are highly adherent and prefer to remain attached to any available substrate rather than existing in suspension in the culture media. Our results showed reduced cellular invasion in COLXV-expressing cells in comparison to the vector controls (Fig. 1E). These data were quantified as the relative percentage of invasion (Fig. 1F) and demonstrate that COLXV expressing cells have reduced invasion in the presence of COLI. Equivalent results were seen when using gelatin, COLI, COLIV and laminin (data not shown). The difference in invasion in cells that overexpressed COLXV could not be attributed to clonal variation in proliferation since growth rates were seen to be independent of whether cells carried the vector or the COLXV plasmid (Fig. 1D).

### Collagen XV Inhibits Collagen I-activated Scatter of BxPC-3 Cells

**Figure 2:**
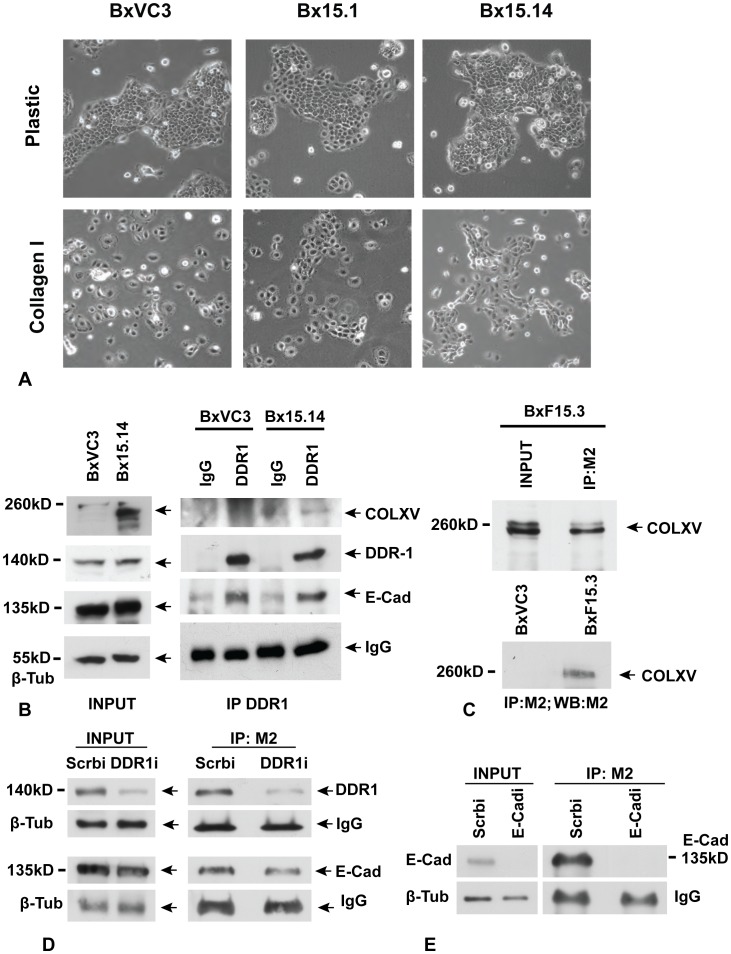
Collagen XV inhibits scatter of BxPC-3 cells on a collagen I substrate and interacts directly with DDR1 and E-Cadherin. A) Vector control (BxVC3) and COLXV expressing (Bx15.1, Bx15.14) clones are shown grown on plastic substrate and on COLI coated substrate. Phase contrast microscopy, all panels 100X magnification. 3 vector clones and 5 COLXV expressing clones (2 with low expression and 3 with high expression) were analyzed at least 3 times and the results were consistent. B) Immunoprecipitation (IP) of DDR1 from vector clone (BxVC3) and COLXV clone (Bx15.14), followed by probing of western blots of the IP material with antibodies specific for COLXV, DDR1 and E-Cad. C) IP of FLAG-tagged COLXV (FCOLXV) from the BxF15.3 clone using the M2 antibody. D) IP of COLXV from clone Bx15.3 with M2 after depletion of DDR1 with a specific siRNA (DDR1i) or transfection of scrambled control siRNA (Scrbi). Depletion of DDR1 reduced the amount of DDR1, but not E-Cad, interacting with COLXV. E) IP of COLXV from clone BxF15.3 with M2 after depletion of E-Cad with a specific siRNA (E-Cadi) or transfection of scrambled control siRNA (Scrbi). Depletion of E-Cad reduced the amounts interacting with COLXV. All experiments were performed a minimum of 3 times with consistent results.

BxPC-3 cells were previously shown to upregulate N-Cad and scatter, when plated on a COLI substrate. Scattering is an *in vitro* process that mimics epithelial to mesenchymal transition (EMT) during tumor progression [37], [38]. Since COLXV is lost from the basement membrane zone prior to metastasis of several tumor types, we asked whether it might interfere with COLI-mediated scatter and thus prevent EMT. BxPC-3 clones expressing COLXV and vector-only control clones were plated on untreated plastic or on COLI-coated plastic and grown for 48 to 72 hours. As observed previously [19], vector control clones scattered on the COLI substrate; however, in contrast, high levels of COLXV expression (Bx15.14, Bx15.24) inhibited the scatter response (Bx15.14 shown in Fig. 2A). Moreover, this effect appeared to correlate with COLXV expression levels since clones Bx15.1 (shown in Fig. 2A) and Bx15.40, which have very little COLXV (Fig. 1A), showed minimal inhibition of scatter.

### DDR1 and E-Cadherin Interact with Collagen XV

To investigate further the mechanism that could explain the effect of COLXV on the scatter response, we next evaluated potential interacting proteins. COLI-mediated activation of BxPC-3 cell scatter is dependent on its simultaneous interaction with DDR1 and α_2_β_1_ integrin [19] so we first examined DDR1. DDR1 is known to suppress integrin signaling through inhibition of Cdc42 activity [39]. DDR1 may also stabilize E-Cad at the cell surface relieving this inhibition of the integrin pathway [24]. To determine whether COLXV is directly interacting with these receptors, we immunoprecipitated DDR1 and then looked for co-immunoprecipitation (co-IP) of E-Cad and COLXV by probing western blots of immunoprecipitated material. DDR1 and E-Cad were shown to be in complex while there was a weak interaction between COLXV and DDR1 (Fig. 2B). Expression of COLXV in BxPC-3 cells had no effect on expression levels of DDR1 or E-Cad proteins (Fig. 2B). To determine whether the COLXV:DDR1 interaction was real, an N-terminal FLAG-epitope tagged COLXV construct (FCOLXV) was transfected into BxPC-3 cells and several high expressing clones generated (clone BxF15.3 is shown in Fig. 2C). COLXV was immunoprecipitated from clone BxF15.3 with the M2 anti-FLAG epitope antibody and Western blots probed with an anti-DDR1 antibody demonstrating that DDR1 is in complex with COLXV (Fig. 2D). To provide further evidence for this interaction, specific siRNAs targeting DDR1 were transfected into the BxF15.3 clone and were shown to diminish the amount of DDR1 in complex with FCOLXV (Fig. 2D). Immunoprecipitation of FCOLXV with M2 followed by probing of western blots with an anti-E-Cad antibody revealed that E-Cad is also in complex with COLXV. Moreover, when siRNAs targeting E-Cad were introduced into the BxF15.3 clone, the COLXV interaction with E-Cad was abolished (Fig. 2E). These data demonstrate a cell-to-stromal complex between DDR1, E-Cad and COLXV, which was confirmed in another pancreatic cancer cell line S2-013. The FCOLXV was stably transfected into S2-013 cells, COLXV immunoprecipitated with the M2 anti-FLAG epitope antibody and Western blots probed with the anti-DDR1 and anti-E-Cad antibodies (Suppl. Fig. S1). Data are shown for clone S2.F15.7 and demonstrate that DDR1 and E-Cad are in complex with COLXV.

### E-Cadherin is Stabilized at the Cell Periphery in Collagen XV Expressing Cells

**Figure 3:**
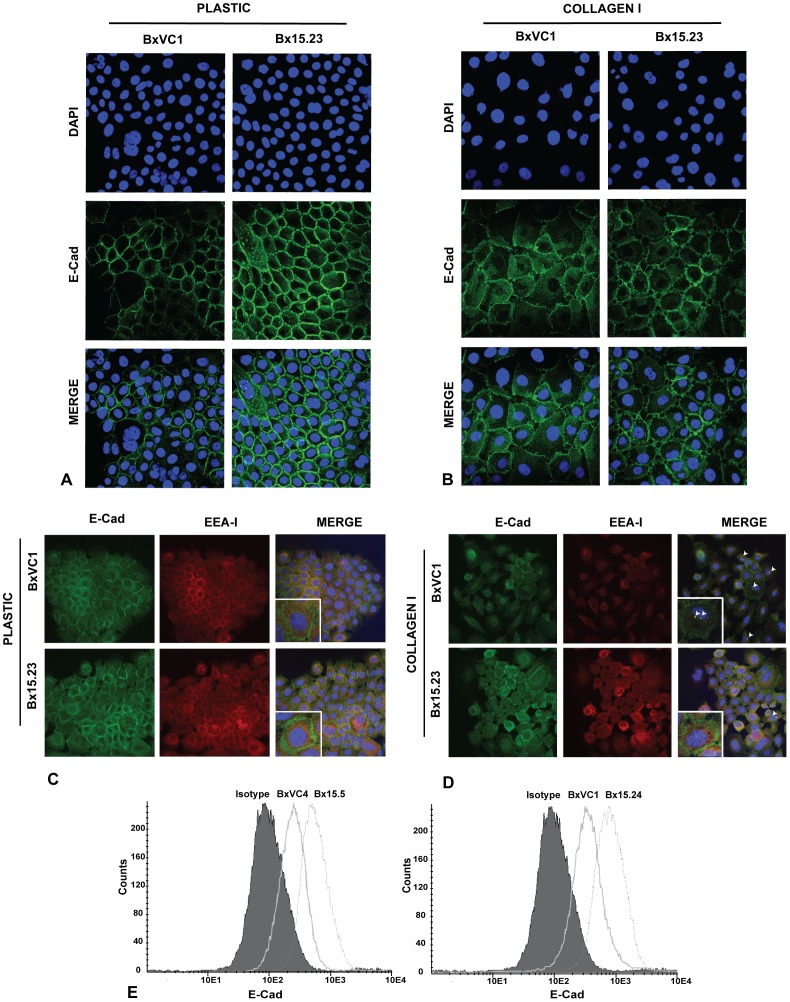
E-Cadherin is stabilized at the cell periphery in collagen XV expressing cells. Confocal microscopy with an antibody specific for the extracellular domain of E-Cad (green) and nuclei stained with DAPI (blue). BxVC1 vector clone and Bx15.23 COLXV clone grown on plastic A) or COLI B). E-Cad is most abundant at the cell surface in both clones on plastic. On COLI, E-Cad moves from the cell periphery into the cytoplasm in BxVC1, but this redistribution is inhibited in the presence of COLXV (BX15.23). C) EEA1 (red) is found in the endoplasmic reticulum (ER)/Golgi zone of the cells grown on plastic, while E-Cad is at the cell periphery. D) After relocation of E-Cad on COLI, EEA1 colocalizes with E-Cad (white arrowheads) in BxVC1 cells but not Bx15.23 cells. Images are representative of several clones. E) Flow cytometry after staining cells with an E-Cad antibody shows increased cell-surface expression of E-Cad in cells with COLXV (Bx15.5 and 15.24) in comparison to vector controls (BxVC4 and BxVC1). All experiments performed a minimum of 3 times with consistent results.

Since DDR1 and E-Cad were shown previously to be in complex [24] and we demonstrate here that COLXV is in complex with both receptors, we next asked whether COLXV stabilized these receptors at the cell surface. When BxPC-3 cells are grown on plastic substrate E-Cad is largely cell surface-associated but becomes cytosolic when the cells scatter on COLI. We demonstrated above (Fig. 2A) that COLXV inhibits this scatter on COLI. Using confocal microscopy, we next observed that COLXV expressing BxPC-3 cells grown on a COLI substrate showed marked stabilization of E-Cad at the cell surface in comparison to the punctate, cytosolic distribution seen in the vector only cells (Fig. 3A, B). Moreover, colocalization of Early Endosome Antigen I (EEAI) with E-Cad when vector- control cells were grown on COLI demonstrated endocytosis and potential recycling of E-Cad to early endosomes (Fig. 3C, D). In contrast, COLXV expressing BxPC-3 cells showed no distinct colocalization of E-Cad and EEAI on COLI and the distribution of these two markers, appeared similar to cells grown on plastic.

E-Cad is expressed at high levels in BxPC-3 cells (Fig. 3A) where its promoter is unmethylated [40]. To determine whether COLXV expression in BxPC-3 cells alters cell surface levels of E-Cad irrespective of the substrate, non-permeabilized cells were stained with an antibody specific for the extracellular domain of E-Cad and subjected to fluorescent activated cell sorting (FACS) analysis. Multiple cell clones expressing COLXV showed increased amounts of E-Cad at the cell surface, (as shown by a shift in the FACS profile), when compared to vector control cell lines (Fig. 3E). These data suggest that E-Cad is localized and stabilized at the cellular periphery in the presence of COLXV.

### Collagen XV Alters Signaling Events Downstream of Cell Surface Receptors for Collagen I

**Figure 4:**
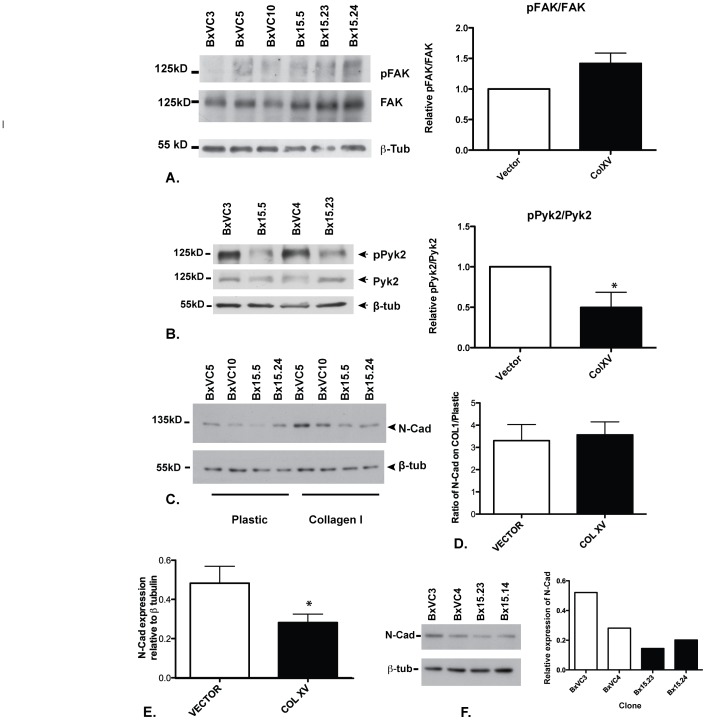
Collagen XV inhibits phosphorylation of Pyk2 and suppresses N-Cadherin. Vector-only (BxVC3, BxVC4, BxVC5, BxVC10) and COLXV-expressing clones (Bx15.5, Bx15.14, Bx15.23, Bx15.24) were grown on COLI coated plates (A, B,); plastic and COLI (C) or plastic alone (F) and lysed after 48 h. Western blots probed with antibodies specific for A) total FAK or pFAK; B) total Pyk2 or pPyk2; C, F) N-Cad; are shown. ß-tubulin provides an estimate of total protein. Expression of COLXV slightly enhances pFAK relative to FAK (though this is not statistically significant, p = 0.0913), but depresses pPyk2 relative to Pyk2 (* p = 0.0447) and suppresses N-Cadherin levels. Blots were quantified by integrating data acquired with the Lasso tool of Adobe Photoshop (C, D, E, F) and analyzed by students t test (E, *p<0.01). Each clone was analyzed at least 3 times for FAK, pFAK, Pyk2, pPyk2 and N-Cad expression.

COLI interacts with cell surface integrins and receptor tyrosine kinases, specifically α_2_β_1_ integrin and DDR1 to stimulate the EMT response [19]. To investigate the impact of COLXV on these signaling pathways, which may be responsible for its inhibition of the scatter response (EMT) and E-Cad stabilization, we next investigated the phospho-signaling pathways downstream of α_2_β_1_ integrin (FAK) and DDR1 (Pyk2). BxPC-3 cells overexpressing and secreting COLXV or vector alone were grown on a COLI substrate and western blots of cell lysates probed with antibodies to total and phosphorylated forms of FAK and Pyk2, the adaptor proteins immediately downstream of α_2_β_1_ integrin and DDR1, respectively. A minor increase in phospho-FAK (pFAK) was seen in comparison to total FAK in the presence of COLXV (Fig. 4A), indicative of pathway activation. These data were confirmed in several BxPC-3 clones expressing COLXV in comparison to vector only clones, however, this change did not reach statistical significance (p = 0.0913). Next, we examined the phosphorylation status of Pyk2 in these clones under the same experimental conditions and observed a significant reduction (p = 0.0447) in Pyk2 phosphorylation (pPyk2) (Fig. 4B) in comparison to total Pyk2, which is indicative of pathway inhibition. Western blots were subjected to densitometry and the signals quantified using the Lasso tool of Adobe Photoshop Extended CS5. In all cases total protein levels were quantitated by probing Western blots with β-tubulin.

COLI-mediated cell scatter in BxPC-3 cells is accompanied by upregulation of N-Cad [19]. To evaluate whether this increase in N-Cad levels was influenced by expression of COLXV, BxPC-3 cells were plated on plastic or COLI substrate for 48 hours and N-Cad measured by western blot of cell lysates, relative to total protein (quantified with an antibody specific for β-tubulin on the same blot). Vector control clones demonstrated scatter on the COLI substrate and N-Cad protein expression was increased as expected (Fig. 4C, D). Despite the ability of COLXV to attenuate the scatter response, BxPC-3 clones expressing the protein showed equivalent levels of N-Cad upregulation on COLI (Fig. 4C, D). However, on plastic substrates we observed a depletion of N-Cad protein in all BxPC-3 clones expressing COLXV in comparison to vector controls (Fig. 4C, 4F). Hence though the relative upregulation of N-Cad on COLI was equivalent in the presence or absence of COLXV (Fig. 4D), the total amounts of N-Cad were less in the former clones (Fig. 4E, 4F).

## Discussion

In previous work, we demonstrated that COLXV altered the growth properties of cervical carcinoma cell lines *in vitro* and was a potent inhibitor of tumor growth from the same lines in nude mice [41]. Moreover, COLXV increased the adhesion of these cells to COLI substrates, a property that was dependent on the N-terminus and collagenous domains of COLXV and was disrupted by mutations in cysteine residues thought to be critical for intermolecular interactions of the protein [10]. These observations led us to investigate the mechanism whereby COLXV acts as a tumor suppressor and to determine whether this might result from COLXV interfering with COLI-mediated pathways of tumorigenesis.

One pathway for COLI activation of EMT is well characterized in the BxPC-3 pancreatic adenocarcinoma cell line [19], [42]. In these cells, COLI binding simultaneously to cell-surface DDR1 and α_2_β_1_ integrin activates downstream signaling pathways that ultimately upregulate N-Cad expression and cause cell scatter. This is accompanied by the redistribution of E-Cad from the cell periphery to the interior, another characteristic event in EMT. Hence we chose to examine the effect of COLXV on COLI activation of EMT in BxPC-3 cells. It is relevant to note that in our experiments the COLXV is secreted from the stably transfected cell clones into the culture medium, where it can interact with COLI and with receptors on the surface of the BxPC-3 cells.

We first demonstrated that overexpression of COLXV impaired the invasion of BxPC-3 cells across COLI-coated substrates. Next, we determined the effect of overexpression of COLXV in BxPC-3 cells on COLI-mediated cell scatter. In comparison to vector-transfected clones that scattered on COLI substrate as observed in the parental cell line [19], overexpression of COLXV reproducibly attenuated this response. Consistent with this result, overexpression of COLXV apparently stabilized E-Cad at the surface of BxPC-3 cells grown on a COLI substrate, and prevented the internalization of E-Cad that accompanies EMT. Since these data suggested that COLXV was somehow interfering with COLI-mediated cell-scatter in BxPC-3 cells, we next used co-immunoprecipitation to look for a direct interaction between COLXV and DDR1, a critical receptor for COLI at the cell surface. We observed that not only is COLXV a ligand for DDR1 and inhibits the DDR1 pathway, it also associates directly with E-Cad. The interaction between COLXV with both DDR1 and E-Cad was confirmed in a second pancreatic adenocarcinoma cell line, S2-013. (This line does not demonstrate COLI-mediated scatter and so was not suitable for additional confirmatory experiments). These data identify the probable signaling pathways responsible for the role of COLXV as a tumor suppressor.

COLXV is normally found within basement membrane zones in proximity to the collagen network (mainly COLI and COLIV) [43] and causes increased adhesion of cells when plated on COLI substrate [10]. This proximity may be critical for the dynamic interplay between the extracellular matrix and the tumor cells. In BxPC-3 cells, the DDR1 signaling pathway, which is activated by COLI [44], and known to be involved in malignancy, is inhibited by exposure to COLXV, suggesting that COLXV interrupts the COLI-mediated scatter (EMT) response. In contrast, activation of α_2_β_1_ integrin-mediated signaling through FAK is enhanced by the presence of COLXV. Since COLI activates both Pyk2 and FAK signaling pathways to cause scatter in BxPC-3 pancreatic adenocarcinoma cells (Suppl. Fig. S2), the functional effect of COLXV on this pathway may be mediated by the imbalance it generates on downstream signaling events.

Also relevant to the mechanism of action of COLXV is the observation that chronic exposure of BxPC-3 cells to high levels of COLXV suppressed the basal levels of N-Cad. Thus, though cells expressing COLXV showed an increase in N-Cad protein when transferred from plastic to a COLI substrate, the amounts of N-Cad were similar to those seen in vector control clones grown on plastic, and substantially less than in vector only cells on COLI. These levels are apparently insufficient to maintain the scatter (mesenchymal) phenotype. The mechanism whereby continuous exposure of BxPC3 cells to extracellular COLXV suppresses N-Cad expression is of interest, since it might provide novel therapeutic targets for pancreas cancer therapy. The simplest explanation is that COLXV binding to DDR1 inhibits Pyk2 phosphorylation and this in turn alters the balance of Pyk2 and FAK phosphorylation that is required for normal activation of N-Cad synthesis. However, there are other possible explanations: COLXV may be attenuating key growth factors such as TGFβ that are known to activate EMT by altering gene expression patterns, via a number of different transcription factors (reviewed in [45]). Alternatively, COLXV may interact with and stabilize other cell surface receptors (in addition to E-Cad) that maintain the pancreatic cells in a more epithelial phenotype and inhibit progression to a mesenchymal state. It is also possible that COLXV may alter other growth properties of the BxPC-3 cells, as we observed for D98 AP2 cervical carcinoma cells [41], though we observed no alterations in cell phenotype (data not shown) or replication rate in BxPC-3 cells expressing COLXV.

Stabilization of E-Cad at the cell periphery in the presence of COLXV provides strong evidence that the tumor cells are retaining their epithelial phenotype. Moreover, the direct interaction between E-Cad, DDR1, and COLXV suggest that COLXV may be part of a complex protein interactome, which may be critical for normal basement membrane function (Suppl. Fig. S2). COLXV could be the tethering factor retaining the integrity of cell polarity and adhesion. Hence, its loss would compromise cellular structure and signaling events, leading to a microenvironment permissive for motile invasive cancer cells to transgress the basement membrane and enter the lymphatic and capillary systems.

## Conclusions

Here we show that extracellular COLXV alters the invasive properties of pancreatic cancer cells and inhibits COLI-mediated cell scatter. We demonstrate that COLXV interacts directly with DDR1 and E-Cad and modulates the signaling pathways that upregulate N-Cad during EMT. Moreover, we show a critical role for collagen XV in E-Cad stabilization at the cell surface, which in turn inhibits EMT and tumor progression.

## Supporting Information

Figure S1 **COLXV is in complex with DDR-1 and E-Cad in S2-013 cells.**
(TIF)

Figure S2 **Schematic model to show possible mechanism of action of COLXV at the surface of pancreatic cancer cells.**
(TIF)
