# Supplementary figures and images for: Collagen XV Inhibits Epithelial to Mesenchymal Transition in Pancreatic Adenocarcinoma Cells
Source: PLoS One. 2013 Aug 22;8(8):e72250. doi: 10.1371/journal.pone.0072250 (PMC3750028; doi:10.1371/journal.pone.0072250)

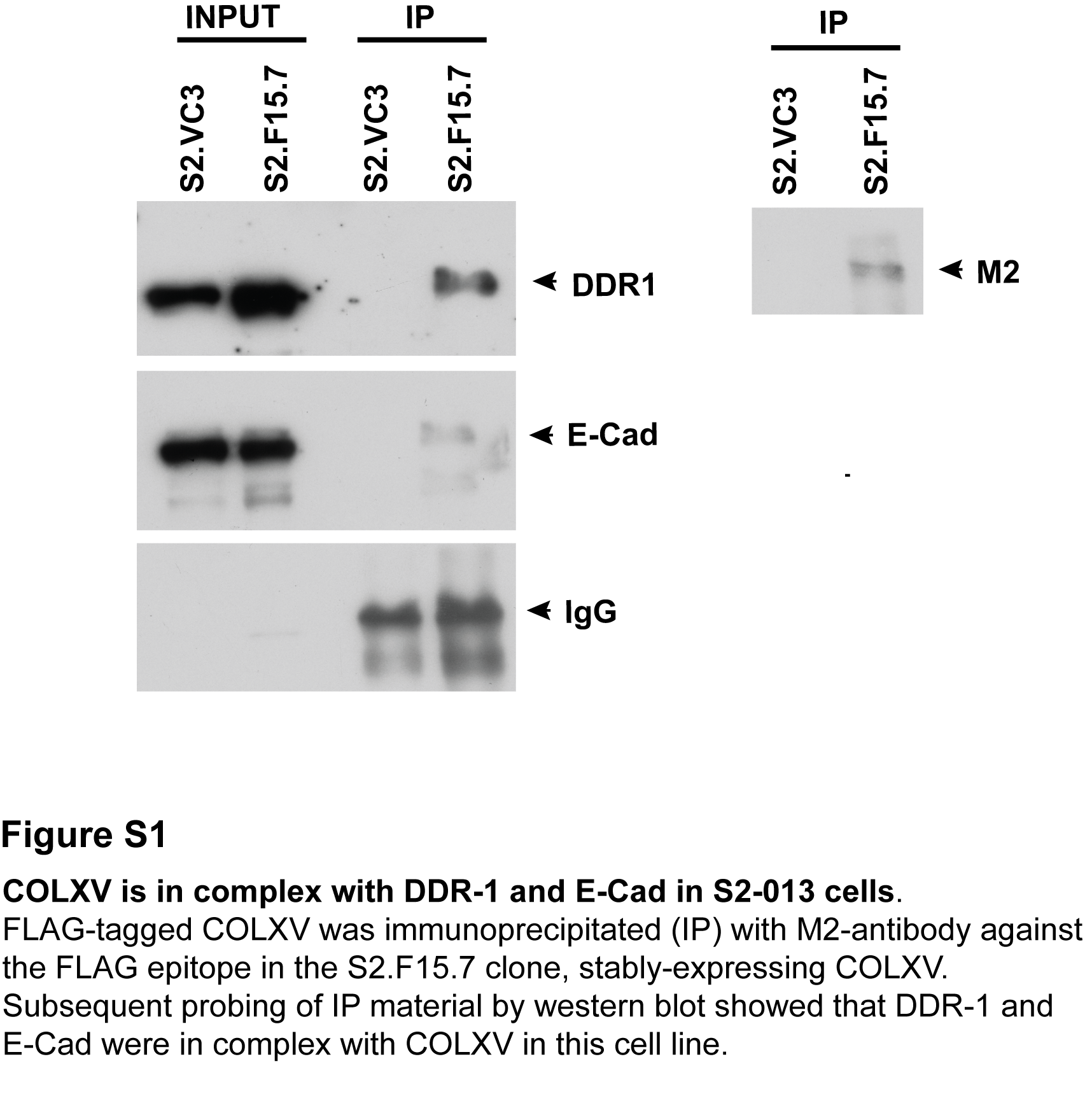

Supplement: Figure S1 — COLXV is in complex with DDR-1 and E-Cad in S2-013 cells. (TIF) [file pone.0072250.s001.tif]

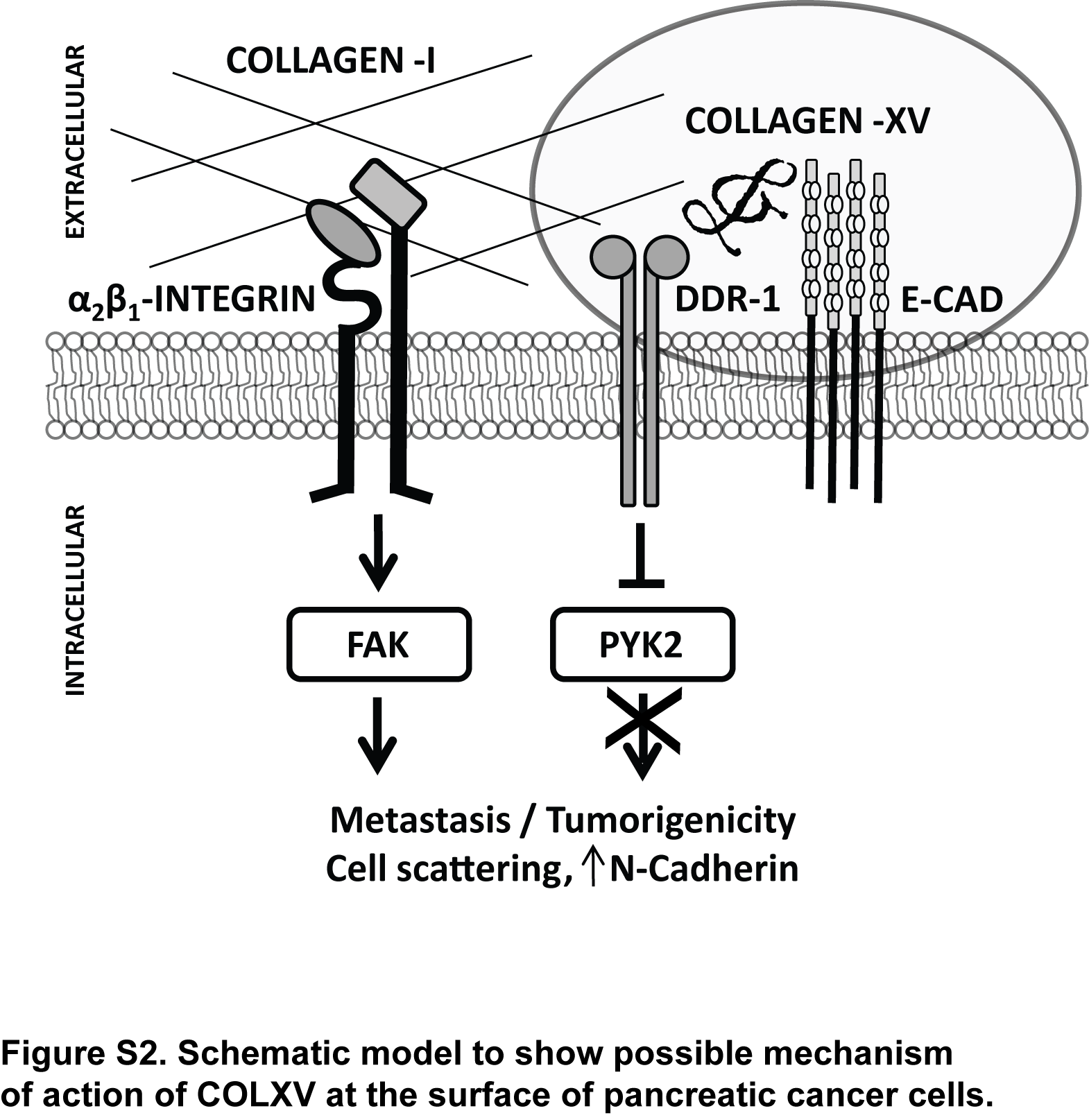

Supplement: Figure S2 — Schematic model to show possible mechanism of action of COLXV at the surface of pancreatic cancer cells. (TIF) [file pone.0072250.s002.tif]
